# *In Vitro* Antimicrobial Activity of Essential Oil of* Thymus schimperi*,* Matricaria chamomilla*,* Eucalyptus globulus*, and* Rosmarinus officinalis*

**DOI:** 10.1155/2016/9545693

**Published:** 2016-01-04

**Authors:** Awol Mekonnen, Berhanu Yitayew, Alemnesh Tesema, Solomon Taddese

**Affiliations:** ^1^College of Medicine, Institute of Medicine and Health Science, Debre Birhan University, P.O. Box 445, Debre Berhan, Ethiopia; ^2^College of Natural Science, Department of Biology, Debre Birhan University, P.O. Box 445, Debre Berhan, Ethiopia

## Abstract

In this study, the* in vitro* antimicrobial activities of four plant essential oils (*T. schimperi*,* E. globulus*,* R. officinalis*, and* M. Chamomilla*) were evaluated against bacteria and fungi. The studies were carried out using agar diffusion method for screening the most effective essential oils and agar dilution to determine minimum inhibitory concentration of the essential oils. Results of this study revealed that essential oils of* T. schimperi*,* E. globulus*, and* R. officinalis* were active against bacteria and some fungi. The antimicrobial effect of* M. chamomilla* was found to be weaker and did not show any antimicrobial activity. The minimum inhibitory concentration values of* T. schimperi* were <15.75 mg/mL for most of the bacteria and fungi used in this study. The minimum inhibitory concentration values of the other essential oils were in the range of 15.75–36.33 mg/mL against tested bacteria. This study highlighted the antimicrobial activity of the essential oil of* E. globulus*,* M. chamomilla*,* T. Schimperi*, and* R. officinalis*. The results indicated that* T. schimperi* have shown strong antimicrobial activity which could be potential candidates for preparation of antimicrobial drug preparation.

## 1. Introduction

Infectious diseases represent a continuous and increasing threat to human health and welfare. They are the major causes for enormous morbidity and mortality in all parts of the world, although developing countries are carrying the major part of the burden [1]. In addition to the usual infectious diseases, incidences of nosocomial and opportunistic infections have risen dramatically. The number of infections caused by new, reemerging, or drug resistant pathogens is growing day by day, and the increased proportion of hospitalized patients with immunodeficiency has resulted in an increase of severe and invasive infections [2]. In order to fill such gaps, new antimicrobial agents are urgently needed.

Plant-derived drugs remain an important resource, especially in developing countries, to combat serious diseases. Approximately 60–80% of the world's population still relies on traditional medicines for the treatment of common illnesses [3, 4]. In Ethiopia, not only do traditional remedies represent part of the struggle of the people to fulfill their essential drug needs but also they are integral components of the cultural beliefs. 80% of the Ethiopian population still depends on traditional medicine [5].

Essential oils do have a plethora of medicinal values [6]. The essential oil from leaves of* Thymus schimperi* possesses anthelmintic, antibacterial, and antifungal activity [7];* Eucalyptus globulus* leaves oil can boost the immune system and is helpful in cases of chicken pox, colds, flu, measles, and infectious diseases [8, 9];* Matricaria chamomilla* flower oil is good for skin care, acne, allergies, boils, burns, eczema, inflamed skin conditions, and wounds and used for infection [10, 11];* Rosmarinus officinalis* leaves oil possesses antitumor and anti-inflammatory actions and antimicrobial activity [12, 13].

In Ankober, northern Ethiopia medicinal plants have been used as traditional medicine to treat different human and livestock ailments by the local people from time immemorial. However, there is no study on the* in vitro* as well as* in vivo* antimicrobial activity of* Thymus schimperi*,* Matricaria chamomilla*,* Eucalyptus globulus*, and* Rosmarinus officinalis* against common microbes. Therefore, this study focuses on* in vitro* antimicrobial activity of essential oil of* Thymus schimperi*,* Matricaria chamomilla*,* Eucalyptus globulus*, and* Rosmarinus officinalis* against selected strains of bacteria and fungi.

## 2. Materials and Methods

### 2.1. Chemicals and Reagents

The following chemicals, solvents, and drugs were used: Mueller-Hinton agar, nutrient broth, MacConkey agar, nutrient agar, methanol, sterile borer, disk diffusion inhibitor zone reading ruler, gentamicin, chloramphenicol, dimethyl sulfoxide, Desiccators, Sabouraud dextrose agar (SDA), potato dextrose agar (PDA), glove, 96% ethanol, liquid soap, and sodium sulphate.

### 2.2. Bacteria and Fungi

The bacterial test microorganisms used in this investigation were* Salmonella typhi*,* Salmonella paratyphi*,* Salmonella typhimurium*,* Shigella species*,* Pseudomonas aeruginosa*,* Staphylococcus aureus*, and* Escherichia coli* and four fungi (two* Trichophyton* spp. and two *Aspergillus* spp.), which were donated by the Ethiopian Public Health Institute (EPHI).

### 2.3. Collection of Plant Material

The fresh leaves of* Thymus schimperi* were collected from Kundi (Ankober District). The fresh leaves of* Rosmarinus officinalis* (rosemary),* Eucalyptus globulus*, and the matured flower heads of* Matricaria chamomilla* were collected from Ankober District and the identity of the plant specimen was confirmed by the National Herbarium in Addis Ababa University, Ethiopia.

### 2.4. Preparation of Essential Oil

The fresh leaves of* Rosmarinus officinalis* (rosemary),* Thymus schimperi*, and Matured Flower head of* Matricaria chamomilla* were dried under shade.* Thymus schimperi* (30 g) was taken into a 1000 mL round bottomed flask and 400 mL distilled water was added to it and then it was distilled by Clevenger-type apparatus for 2 hr [14].* Rosmarinus officinalis* (30 g) was taken in to a 1000 mL round bottomed flask and 300 mL distilled water was added to it and then it was distilled by Clevenger-type apparatus for about 3 hr to get colorless oil [14].* Matricaria chamomilla* (30 g) was taken in to a 1000 mL round bottomed flask and 400 mL distilled water was added to it and then it was distilled by Clevenger-type apparatus for 4 hr [14]. The crude oil obtained was transferred into a separatory funnel and the oil was separated from the upper layer. The oil was then dried over anhydrous sodium sulphate and stored at −4°C before analysis.

The fresh leaves of* Eucalyptus globulus* were completely immersed in 1000 mL round bottomed flask with distilled water and then hydrodistilled in a full glass Clevenger-type apparatus to give colorless oil. This process was continued for 3 hr. The oil was allowed to stand for sufficient time, to be clear, and then it was collected carefully after draining out condensed water. The oil was then dried over anhydrous sodium sulphate and stored at −4°C until use [14].

### 2.5. The Antimicrobial Assays

#### 2.5.1. Preparation of Inoculums

The bacteria used for the study were prepared by inoculating isolates into nutrient broth and incubated at 37°C for 24 hr. Fungal strains used for the study were prepared by inoculating isolates into SDA/PDA and incubated at 30°C.

#### 2.5.2. Antimicrobial Sensitivity Testing

Agar well diffusion method was used to determine zone of inhibition. Mueller-Hinton agar for bacteria, SDA for* Trichophyton *spp., and PDA for* Aspergillus *spp. were used. About 20–25 mL of molten medium cooled to 45°C and was added to presterilized plates (150 mm in size). After these 16–24-hour-old cultures of bacterial species, 48-hour-old cultures of* Aspergillus* spp. and 7-day-old cultures of* Trichophyton* spp. were spread using a sterile cotton swab and each microbe evenly spread over the entire surface of agar plate to obtain a uniform plate surface growth. Petri plates were allowed to dry. About 3-4 wells in each plate of 6 mm diameter and 5 mm depth were punched in agar surface with the help of a sterilized borer for placing the extracted oil samples. About 50 *μ*L of the undiluted essential oil of each plant was dispensed into respective wells and 10 mcg gentamicin was used as a positive control. Dimethyl sulfoxide (DMSO) was used as negative control. The plates were then left at room temperature for 30 minutes and then incubated at 30°C for 7 days for* Trichophyton* spp. and plates with* Aspergillus* for 48 hours and plates with bacteria were incubated for 24 hours at 37°C. After incubation, the zones of inhibition were measured using a ruler and the results reported in millimeters (mm). All the tests were run in triplicate and the average result was taken [15, 16].

#### 2.5.3. Determination of Minimum Inhibition Concentration (MIC)

Minimum inhibition concentration was determined using the agar dilution method. The MIC was evaluated on plant extracts that showed antimicrobial activity in the agar well diffusion assay on any organism. This test was performed at five concentrations of each extract (250 mg/mL, 125 mg/mL, 62.5 mg/mL, 31.25 mg/mL, and 15.75 mg/mL) employing doubling serial dilutions of plant extracts in nutrient broth up to the fifth dilution. Overnight incubated suspension of each organism in nutrient broth was prepared and 50 *μ*L was added to all the test tubes and preparations were incubated at 37°C for 24 hours. After incubation, using a sterile cotton swab, suspension of each tube was inoculated on nutrient agar to see if bacterial growth was inhibited or not. Growth of bacteria on solid media indicated that a particular concentration of extract was unable to inhibit the bacteria. The MIC was defined as the lowest concentration of an antimicrobial that inhibited the visible growth of a microorganism after overnight incubation [17].

### 2.6. Data Analysis

All the measurements were replicated three times for each assay and the results are presented as mean ± SD. Data was analyzed using windows SPSS version 17.0 and descriptive statistic was used.

## 3. Result

### 3.1. Essential Oil Analysis

The main constituents of the essential oil of the tested four plants identified by GC/MS are presented in Table 1 according to their percentage composition [14].

### 3.2. Antibacterial Activity

#### 3.2.1. Inhibition Zone Determination

**Figure 1:**
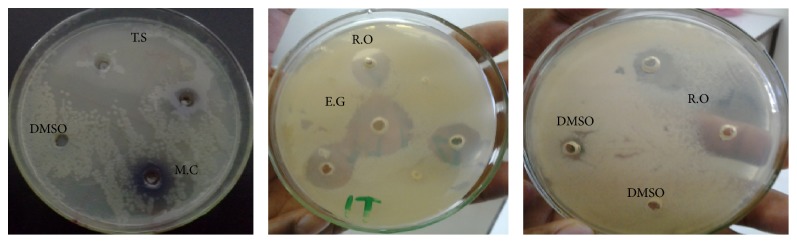
Growth inhibition zone on* S. aureus*. (T.S =* Thymus schimperi*, E.G =* Eucalyptus globulus*, R.O =* Rosmarinus officinalis*, M.C =* Matricaria chamomilla*, and DMSO = Negative control.)

The essential oils tested exhibited different degrees of antibacterial activity against tested bacterial pathogens except* Matricaria chamomilla* essential oil, which failed to inhibit growth of the tested bacterial pathogens (Figure 1). Overall essential oil of* T. schimperi*,* E. globulus*, and* R. officinalis* showed comparable inhibition of growth of the bacteria.* T. schimperi* exhibited almost the highest activity against all the tested bacteria measured in the range of 12–33 mm as shown in (Table 2).* T. schimperi* has shown the greatest inhibition zone diameter of 33 mm against* S. epidermidis*. The oils of* E. globulus* exhibited significant antibacterial activity as observed by its inhibition zone of 10–32 mm. The oils of* R. officinalis* also inhibited the growth of the tested bacteria and its zone of inhibition was 6–32 mm. As to the standard drugs used in the test, the inhibition zone for Gentamicin was 18–21 mm. Gentamicin failed to inhibit the growth of* Shigella* spp. The negative control DMSO showed no inhibition of the growth of tested bacteria.

**Figure 2:**
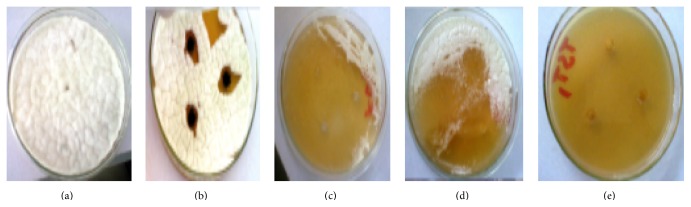
Growth inhibition zone on* Trichophyton* spp1 ((a) = DMSO, (b) =* Matricaria chamomilla*, (c) =* Rosmarinus officinalis*, (d) =* Eucalyptus globulus*, and (e) =* Thymus schimperi*).

**Figure 3:**
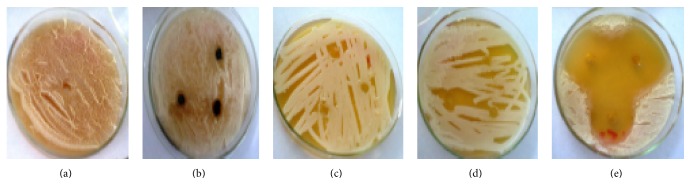
Growth inhibition zone on* Trichophyton* spp2 ((a) = DMSO, (b) =* Matricaria chamomilla*, (c) =* Rosmarinus officinalis*, (d) =* Eucalyptus globulus*, and (e) =* Thymus schimperi*).

**Figure 4:**
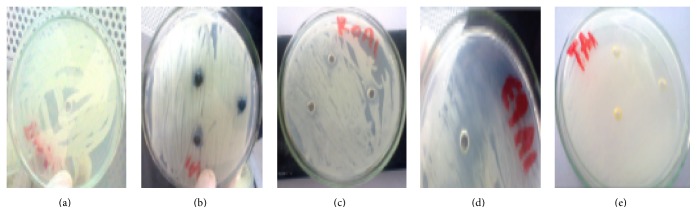
Growth inhibition zone on* Aspergillus* spp1 ((a) = DMSO, (b) =* Matricaria chamomilla*, (c) =* Rosmarinus officinalis*, (d) =* Eucalyptus globulus*, and (e) =* Thymus schimperi*).

**Figure 5:**
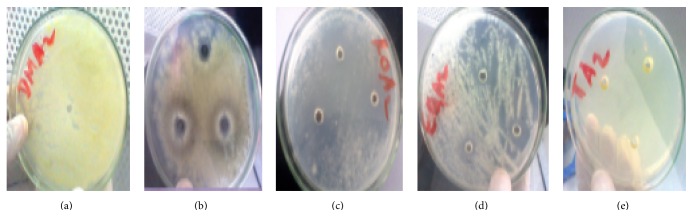
Growth inhibition zone on* Aspergillus* spp2 ((a) = DMSO, (b) =* Matricaria chamomilla*, (c) =* Rosmarinus officinalis*, (d) =* Eucalyptus globulus*, and (e) =* Thymus schimperi*).

As shown in Table 3, all the oils tested exhibited different degrees of antifungal activity against two* Trichophyton *spp. (Figures 2 and 3) and two* Aspergillus *spp. (Figures 4 and 5). Of all the different essential oils tested, the maximum antimycotic activity was shown by* T. schimperi* that inhibit the growth of all tested fungi.* T. schimperi* has shown the greatest inhibition zone diameter.* E. globulus* and* R. officinalis* exhibited antifungal effects against one or more microorganisms.* Trichophyton *spp1 was inhibited by both* E. globulus* and* R. officinalis* with zones of inhibition of 27.3 and 28.6 mm, respectively. However,* Trichophyton *spp2 was not sensitive to both these essential oils. The oils of* E. globulus* and* R. officinalis* exhibited moderate activity against* Aspergillus *spp. On the other hand,* M. chamomilla* did not inhibit the growth of all tested fungi. The control DMSO showed no inhibition. As indicted in Table 3,* T. schimperi* inhibited the growth of all tested fungi at a concentration lower than 15.75 mg/mL.

#### 3.2.2. Minimum Inhibitory Concentration

MIC of essential oil of* T. schimperi*,* E. globulus*, and* R. officinalis* is presented in Table 4. Since* Matricaria chamomilla* essential oil failed to inhibit the growth of tested bacterial pathogens, MIC determination assay was not conducted. On the basis of the results obtained,* T. schimperi* revealed remarkable antibacterial effect.* T. schimperi* essential oil inhibited the growth of almost all of tested bacteria at concentration lower than 15.75 mg/mL. However, it needs higher concentration (23.25 mg/mL) to inhibit growth of* P. aeruginosa*. On the other hand, as can be noted from Table 4,* E. globulus* essential oils inhibited growth of tested bacteria in concentrations 15.75–31.25 mg/mL.* R. officinalis* was not as effective as others; it exhibited less antibacterial activity for tested bacteria with MIC values ranges 15.75–36.33 mg/mL. The lowest concentration of the* R. officinalis* essential oil at which* S. epidermidis* was unable to grow was found to be <15.75 mg/mL which makes it as effective as* T. schimperi*.

## 4. Discussion

Infectious diseases represent a continuous and increasing threat to human health and welfare. Infectious diseases of bacterial origin, such as* S. aureus*,* Salmonella *spp., and* Shigella *spp., constitute the major causes of morbidity and/or mortality in developing countries like Ethiopia [1, 17]. Mycotic infection is also a common disease in developing countries. Although there are various drugs for treating infectious disease, microorganisms develop resistance for most conventional drug therapies. Therefore, there is need for new cost-effective therapies with better efficacy. Medicinal plants are important sources of new chemical substances that have beneficial therapeutic effects. Today, a substantial number of drugs are developed from plants which are active against a number of diseases [18].

In this study, the antimicrobial effect of essential oils from four plants,* T. schimperi*,* E. globulus*,* R. officinalis*, and* M. chamomilla*, was tested against bacteria and fungi. The results in the present study indicate that essential oils extracted by hydrodistillation applied with the same concentration have variable antimicrobial effect against* S. aureus*,* S. pyogenes*,* S. typhi*,* S. epidermidis*,* E. coli*,* Shigella *spp.,* P. aeruginosa*, two* Trichophyton *spp1, and two* Aspergillus *spp.* in vitro*. Among the four plants,* Thymus schimperi* which is endemic to Ethiopia was overall effective against all the test organisms.

One of the models used to study the* in vitro* antimicrobial activity of plant extract is measuring of the inhibition zone. The results of this study showed inhibition of the growth of tested bacteria and fungi as it was noticed by measuring inhibition zone.* Thymus schimperi* caused higher inhibition zone with the range of 12 mm–33 mm against bacteria. The essential oil of this plant was found to be the most active against all the bacteria used in this study. Similarly,* T. schimperi* also has been found to exhibit an overall superiority in its antifungal activity compared to other plant extracts. It is most probably due to thymol, which is the main constituents of the essential oil and which is the compound found to have the widest spectrum of activity against several bacterial and fungal strains [19]. Moreover, carvacrol which exhibits fungicidal, insecticidal, antimicrobial activities and anticarcinogenic and antitumor activities is the main constituent of this oil (Table 1). Therefore, these two components could be responsible for strong antimicrobial activity of* Thymus schimperi*. The result of this study is found to be comparable to other findings which revealed the various inhibitory effect of this oil against* Staphylococcus aureus*,* Pseudomonas aeruginosa*, and* Escherichia coli* [20].

In this study the essential oil extracted from* E. globulus* leaf demonstrated strong antibacterial activity. These results are in line with the report by Bachir and Benali [8], which showed the extracts from* E. globulus* possess antibacterial activity against* E. coli* and* S. aureus*. The antibacterial activity observed in this study could be attributed to *α*-pinene and 1,8-cineole components present in the oil. However, another study by Damjanović-Vratnica et al. [21] showed the antibacterial activity of essential oil of* E. globulus* against tested bacterial strain is higher than antibacterial activity of the present study. The possible reason for observed difference between these two studies could be due to the greater content of 1,8-cineole (85.82%), which is responsible for antimicrobial activity [21]. The percentage composition of 1,8-cineole content of the present study is 63.00% which was less than the range reported by British Pharmacopoeia. The oil exhibited moderate activity against the fungus which is lesser than the expected value. It is probably due to the less amount of 1,8-cineole in the eucalyptus essential oil already known as the component that inhibits the growth of fungi.

Leaves of* R. officinalis* essential oil exhibited antibacterial effect against tested bacteria. This finding agrees with the studies done in other places [22, 23]. The major components of this oil, *α*-pinene, have been known to exhibit antimicrobial activity against the bacterial strains (*Staphylococcus aureus*,* Staphylococcus epidermidis*,* Pseudomonas aeruginosa*,* Shigella flexneri*,* Klebsiella pneumoniae*,* Salmonella typhi*,* Serratia marcescens*, and* E. Coli*) [22]. Essential oils rich in *α*-pinene demonstrated potential antibacterial activity [22, 23]. The bridged bicyclic monoterpenes *α*-pinene and *β*-pinene showed considerable biological activity. On the other hand, enantiomers of *α*-pinene, *β*-pinene, limonene, and linalool have a strong antibacterial activity [23]. The antimicrobial activity revealed that these leaves had similarity to those of other* R. officinalis* essential oils analyzed by Derwich et al. [22], in which the major component was *α*-pinene. Other study revealed the 1.8-cineole, which has been known to exhibit antimicrobial activity against the bacterial strains (*Escherichia coli*,* Pseudomonas aeruginosa*,* Staphylococcus typhi*,* Staphylococcus aureus*,* Staphylococcus intermedius*, and* Bacillus subtilis*) [22, 24]. The oil revealed moderate activity against the fungus. The cineole containing more than 49% of 1,8-cineole showed the highest antifungal activity [25].

On the other hand, flower of* M. chamomilla* essential oil did not show any antibacterial activity at the test concentrations, unlike other essential oils which exhibit antibacterial activity with same concentration. The activity profile of* M. chamomilla* essential oil observed did not agree with previously reported study [26, 27] on this plant which provided antibacterial activity against different bacteria. Other published data revealed that the antimicrobial activity of the essential oil of* M. chamomilla* is apparently related to its *α*-pinene, camphene, sabinene, 1,8-cineole, bisabolol oxide, and *α*-bisabolol components [27]. The reason for lack of antibacterial activity of* M. chamomilla* could be associated with less amount of active component especial *α*-bisabolol (4.139%) which is very low as compared with other published data (56.86%) [28]. Seasonal variation and altitude could be responsible for variability in the amount of secondary metabolites of the plant. Similarly, flower of* M. chamomilla* essential oil did not show any antifungal activity at the test concentrations. Selected compounds of the* M. chamomilla* flower essential oil including *α*-bisabolol, spiroethers, chamazulene, and umbelliferone have been reported to have antifungal activities [27, 28]. The possible mechanism of action of fungal growth inhibition by plant essential oils, probably associated with corresponding morphological alterations in hyphal compartments, may be a consequence of interactions between essential oil components and enzymes involved in cell wall synthesis, which affects fungal growth and morphogenesis. Published data indicated that *α*-bisabolol from* M. chamomilla* may inhibit fungal growth via specific inhibition of ergosterol biosynthesis [29]. As stated above in the present work, the content of *α*-bisabolol was recognized less as compared with other reported data. So the lack of antifungal activity of* M. chamomilla* essential oil against tested fungal may be justified by the less amount of this important component [28].

Determination of MIC value further showed antibacterial activity of essential oil of* T. schimperi*,* E. globulus*, and* R. officinalis*. The MIC was defined as the lowest concentration of antimicrobial that inhibited the visible growth of a microorganism after overnight incubation [17]. MIC values did not exhibit substantial variations when compared to the trend of inhibition shown with the plate diffusion method. Generally, larger inhibition zone values correlated with lower MIC. Among the plants studied, the* T. schimperi* oil has been found to exhibit an overall superiority in its antimicrobial activity compared to other plants as it was evidenced by its lower MIC value. This study also showed that* T. schimperi* can inhibit all tested fungi with very low concentration. This result further strengthens the strong antifungal activity of essential oil of* T. schimperi*.

Overall, it is not surprising that there are differences in antimicrobial activities of these four plant groups due to the variations in type and/or amount of phytochemical constituents present in them. There is difference between data generated from this study as compared with other reported data. This might be explained by the differences in susceptibility testing conditions, method of extraction, species differences, and even strain to strain differences.

## 5. Conclusion

This study highlighted the antimicrobial activity of the essential oil of* E. globulus*,* M. chamomilla*,* T. schimperi*, and* R. officinalis*. The results indicated that* T. schimperi* have shown strong antimicrobial activity which could be potential candidates for preparation of antimicrobial drug preparation. Though, the antimicrobial activities of the* R. officinalis* and* E. globulus* oils are less effective than the* T. schimperi* oils, their antimicrobial activities are not regarded as useless. However, the* M. chamomilla* oil failed to show any antimicrobial activity.

## Figures and Tables

**Table 1:** Chemical composition of essential oils of *Rosmarinus officinalis*, *Thymus schimperi*, *Matricaria chamomilla*, and *Eucalyptus globulus* [14].

*Rosmarinus officinalis*		*Thymus schimperi*		*Matricaria chamomilla*		*Eucalyptus globulus*	
Compound	%	Compound	%	Compound	%	Compound	%
*α*-Pinene	50.830	*α*-Phellandrene	1.062	trans-beta-Farnesene	6.953	*α*-Pinene	16.101
Camphene	5.211	*α*-Pinene	0.213	(Z,Z)-alpha-Farnesene	1.004	*β*-Pinene	0.416
*β*-Pinene	2.068	p-Myrcene	15.250	Germacrene B	0.253	Limonene	1.371
*β*-Myrcene	0.683	*γ*-Terpinene	9.020	*β*-Pinene	0.218	1,8-Cineole	63.001
*α*-Terpinene	0.356	Trans-sabinene hydrate	0.046	1,3,8-para-Menthatriene	0.273	*α*-Phellandrene	1.324
Limonene	1.729	Ocimene	0.051	*α*-Bisabolol oxide B	51.428	Sabinene	1.211
1,8-Cineole	24.425	Linalool	0.159	*α*-Bisabolol	4.139	Camphor	3.422
*γ*-Terpinene	0.674	Thymol	0.104	Chamazulene/azulene	17.688		
*α*-Terpinolene	0.496	Carvacrol	71.024	Limonene	3.298		
Linalool	1.262	Ethanone	1.326	En-in-dicycloether	10.841		
Camphor	3.845	1,2-Benzenediol	0.374	Butanedioyldihydrazide	1.391		
Borneol	1.517	*β*-Sesquiphellene oxide	0.108	*α*-(1-Naphthyl) ethylamine	0.498		
*α*-Terpineol	0.813	Caryophyllene oxide	0.087	Hexadecane	0.230		
Verbenone	0.521			Dotriacontane	0.752		
Bornyl acetate	1.628						
Caryophyllene	2.941						
*α*-Humulene	0.393						

**Table 2:** Antimicrobial activity of the *E. globulus*, *M. chamomilla*, *T. schimperi*, and *R. officinalis* essential oil and standard antibiotics against bacteria.

Bacteria	Mean of zone inhibition in mm (mean ± SD)					
	Plant species				Gentamicin	DMSO
	*T. schimperi*	*E. globulus*	*R. officinalis*	*M. chamomilla*		
*S. aureus *	23.5 ± 1.52	32 ± 1.34	25 ± 1.23	NI	18 ± 1.21	NI
*S. pyogenes *	21 ± 0.87	16 ± 0.9	17 ± 1.11	NI	20 ± 0.91	NI
*S. epidermidis*	33 ± 1.5	20 ± 1.00	32 ± 1.2	NI	21 ± 0.99	NI

*S. typhi *	27.3 ± 1.1	18 ± 0.9	20.3 ± 1.00	NI	18 ± 0.88	NI
*E. coli*	12 ± 0.4	10 ± 0.52	6 ± 0.21	NI	21 ± 1.10	NI
*Shigella* spp.	20 ± 1.2	18 ± 0.89	22 ± 1.3	NI	NI	NI
*P. aeruginosa*	16 ± 1.00	28 ± 1.1	17 ± 1.2	NI	19 ± 0.90	NI

NI: no inhibition.

**Table 3:** Antimicrobial activity of the *E. globulus*, *Matricaria chamomilla*, *T. schimperi*, and *Rosmarinus officinalis* essential oil against fungi.

Fungus	Mean of zone inhibition in mm (mean ± SD)					MIC (mg/mL)
	Plant species				DMSO	*T. schimperi*
	*T. schimperi*	*E. globulus*	*R. officinalis*	*M. chamomilla*		
*Trichophyton *spp1	MI	27.3 ± 1.10	28.6 ± 1.2	NI	NI	<15.75
*Trichophyton *spp2	40 ± 0.00	NI	NI	NI	NI	<15.75
*Aspergillus *spp1	23.7 ± 0.85	11 ± 1.3	NI	NI	NI	<15.75
*Aspergillus *spp2	MI	NI	17 ± 0.6	NI	NI	<15.75

NI: no inhibition; MI: maximum inhibition.

**Table 4:** Minimum inhibitory concentrations (MIC) of *E. globulus*, *T. schimperi*, and *Rosmarinus officinalis* essential oils against the tested bacteria.

Bacteria	Minimum inhibitory concentration (MIC) in mg/mL			
	Plant species			
	*T. schimperi*	*E. globulus*	*R. officinalis*	*M. chamomilla*
*S. aureus *	<15.75	15.75	23.25	N.T
*S. pyogenes *	<15.75	20.58	31.25	N.T
*S. epidermidis*	<15.75	15.75	<15.75	N.T

*S. typhi *	<15.75	20.58	20.58	N.T
*E. coli*	<15.75	23.25	31.25	N.T
*Shigella *spp.	<15.75	20.58	25.91	N.T
*P. aeruginosa*	23.25	31.25	36.33	N.T

N.T: not tested.
